# Cerebral Ischemic Events: An Overlooked Complication of Transthyretin Cardiac Amyloidosis in Afro-Caribbean Patients

**DOI:** 10.3389/fneur.2022.878292

**Published:** 2022-05-19

**Authors:** Rishika Banydeen, Aissatou Signate, Tuan-Huy Tran, Astrid Monfort, Remi Neviere, Jocelyn Inamo

**Affiliations:** ^1^Clinical Research Department, CHU Martinique (University Hospital of Martinique), Fort de France, France; ^2^Cardiovascular Research Team EA7525, Université des Antilles (University of the French West Indies), Fort de France, France; ^3^Department of Neurology, CHU Martinique (University Hospital of Martinique), Fort de France, France; ^4^Department of Cardiology, CHU Martinique (University Hospital of Martinique), Fort de France, France

**Keywords:** transthyretin cardiac amyloidosis, cerebral ischemic event, cardioembolic pattern, atrial fibrillation, Afro-Caribbean

## Abstract

**Aim:**

The link between transthyretin cardiac amyloidosis (CATTR), and cerebral ischemic events (CIE) has only been hinted at till now, impeding progress in patient management. We seek to evaluate the frequency and characteristics of CIE in Afro-Caribbean patients followed for CATTR at our institution.

**Methods:**

In this single-center retrospective observational study, Afro-Caribbean patients followed for CATTR between July 2005 and October 2019 were included. Occurrence of CIE was investigated, and their cardioembolic origin determined. Analysis of patient characteristics was conducted according to CIE and CATTR profiles.

**Results:**

Overall, 120 CATTR patients were included: 17 wild-type ATTR (14.2%), 73 ATTR-V122I (60.8%), and 22 ATTR-I107V (18.3%). Thirty-six patients (30.0%) presented with CIE, including three transient ischemic attacks and 33 permanent ischemic strokes (75.8% with a cardioembolic pattern). CIE was concomitant with CATTR diagnosis in 16 (16/36: 44.4%) patients, while 14 patients (14/36: 38.9 %) experienced CIE over a median CATTR follow-up of 2.0 years (min-max range: 0.8–4.4 years). CATTR-CIE patients presented with atrial fibrillation (66.7%), left atrial enlargement (77.8%), a CHA_2_DS_2_-VASc ≥ 3 (97.2%) and a high anticoagulant intake (75.0%). Multivariate analysis retained only a high CHA_2_DS_2_-VASc score as an independent predictor of CIE risk (Hazard Ratio [95% CI]: 12.03 [1.62–89.24]).

**Conclusion:**

Concomitant CIE, and CATTR diagnosis, potentially carries a worse prognosis. A CHA_2_DS_2_-VASc score ≥3 seems to be a strong and independent predictive factor of CIE in CATTR patients. Further studies are needed to assess the efficacy and timeliness of anticoagulation in CATTR patients, independently of atrial fibrillation.

## Introduction

Systemic amyloidosis is a vast group of diseases defined by the presence of insoluble misfolded autologous protein deposits in various tissues and organs (1). In cardiac forms (cardiac amyloidosis, CA), amyloid fibrils accumulate within the myocardial interstitium leading to restrictive and hypertrophic cardiomyopathies often culminating in symptomatic heart failure and death. Amyloid precursor proteins involved in CA are mainly immunoglobulin light chain (AL) and transthyretin (TTR), with two distinct transthyretin amyloidosis (ATTR) types, namely hereditary or mutated (also known as ATTRv, v for variant) and wild type (ATTRwt) (2–4). Rapidly progressive forms of CA related to TTR variants V122I (p.Val142Ile) and I107V (p.Ile127Val) have been identified in populations of African descent (Afro-Americans, Afro-Caribbeans), with both variants typically associated with either predominantly cardiac or predominantly neuropathic or mixed cardiac-neuropathic phenotypes (5–13).

Central nervous system (CNS) involvement in ATTRv is more and more commonly evoked in the clinical setting. In TTR mutations, such as V30M (p.Val50Met), cerebral amyloid angiopathy (CAA), a disease of the small arteries and arterioles predominantly affecting the cortex and leptomeninges, is primarily characterized by intracerebral lobar and sub-arachnoid hemorrhage, cerebral micro-bleeds and cortical superficial siderosis (14, 15). Moreover, growing evidence now suggests that cerebral ischemic events (CIE) might be a common complication of CA in Caucasians presenting with AL amyloidosis (16, 17). In contrast, only sporadic case series have reported CIE in patients presenting with cardiac transthyretin amyloidosis (ATTR cardiomyopathy, CATTR). Even rarer are reports in patients of African descent (Afro-Americans, Afro-Caribbeans) with CATTR-V122I and CATTR-I107V (17–21).

While hereditary transthyretin amyloidosis remains a rare event (one case/100,000 Caucasians), even in expert medical centers for amyloidosis management worldwide (Sweden, Ireland, Spain, mainland France, Finland, Germany, Greece), it is assumed to be more prevalent in populations of predominantly African descent (22, 23). Our present work aims to analyze the occurrence and characteristics of cerebral ischemic events (CIE) in a real-world cohort of Afro-Caribbean patients followed for CATTR at our institution.

## Patients and Methods

### Study Design and Population

This single-center retrospective observational study is set in the French Caribbean island of Martinique (14.6415° N, 61.0242° W) at the unique University Hospital. Martinique is one of the most highly developed islands in the Caribbean basin, classified high (41st) in terms of global human development at the world level. The population, of 355 094 inhabitants (January 1^st^ 2020), is predominantly of Afro-Caribbean origin (24). The University Hospital of Martinique is equipped with a technical platform in neurology, cardiology and hematology, and hosts the only expert medical center for amyloidosis management of the Caribbean region.

For study purposes, CATTR patients seen at the University Hospital from July 2005 to October 2019 were retrospectively evaluated for CIE occurrence by using multiple overlapping hospital sources. All patients were managed in accordance with the amended Declaration of Helsinki (http://www.wma.net/en/30publications/10policies/b3/) and gave their informed consent for the processing of personal data for scientific research purposes. The study was approved by the hospital's institutional review board. Demographic data, personal medical history, neurological and cardiovascular assessment, ongoing treatments, and follow-up events were collected in a centralized anonymized database. Patient ethnicity was determined via self-reported information collected at the time of hospital registration. Patients' vital status was controlled till May 10^th^ 2021 (time of statistical analysis).

### Diagnosis of TTR Amyloidosis

Patients received a 12-lead ECG with echocardiography and nuclear imaging scintigraphy. CATTR was diagnosed by cardiac uptake on 99mTc-labeled phosphate bone scintigraphy. Cardiac involvement was confirmed by nuclear imaging showing cardiac uptake grade ≥2 of the Perugini classification of bone tracer technetium-99m-labeled hydroxy methylene diphosphonate [(99mTc)-HMDP] (25), cardiac echography revealing the presence of left ventricular hypertrophy (diastolic interventricular septum thickness ≥12 mm on echocardiography without other causes of left ventricular hypertrophy) and decrease in longitudinal global strain with abnormal apical texture characterized as a speckled appearance (1). The diagnosis of TTR amyloidosis was confirmed by histological demonstration of amyloid fibrils in salivary duct gland, subcutaneous adipose tissue or endomyocardial biopsies. TTR amyloidosis phenotypes, i.e., isolated cardiomyopathy or associated with amyloid peripheral neuropathy, were determined. Peripheral neuropathy was assessed on the basis of clinical criteria, associated to electroneuromyogram (ENMG) evaluation. Genetic testing for TTR variant was performed in all CATTR patients.

### Cerebral Ischemic Event (CIE)

All patients were initially evaluated by a senior neurologist with a complete medical history assessment and physical examination. Cerebral ischemic event (CIE) was defined as transient or permanent neurologic impairment and disability due to vascular causes. The presence of a focal neurologic deficit lasting <24 h and normal brain imaging characterized the diagnosis of a transient ischemic attack (TIA) (26). Initial studies included brain computed tomography (CT), magnetic resonance imaging (MRI), routine blood biochemistry and vascular studies of intracranial and extra-cranial arteries (computed tomography angiography, carotid duplex, transcranial Doppler, and angiography). CIE was documented showing hypodensity (CT scan) and/or a hypersignal in T2 FLuid Attenuation Inversion Recovery (FLAIR) sequence and/or imaging diffusion sequence in encephalic magnetic resonance (MRI), systematized in terms of adequate topographical arterial territory (27). Patients with neuropathologic or neuroimaging evidence of probable or possible CAA (evidence of lobar, cortical and cortical-subcortical intraparenchymal hemorrhage, cerebral microbleeds, and/or cortical superficial siderosis in the absence of obvious other pathologies) as well as those with neurologic dysfunction due to non-vascular causes (i.e. brain tumor) were excluded from the present analysis (28). In order to better characterize the etiology of stroke in patients with CATTR, the ASCOD classification (A: atherosclerosis; S: small vessel disease; C: cardiac pathology; O: other causes; D dissection) was used to phenotype ischemic stroke. Cardioembolism was screened using ASCOD definitions of cardiac pathology, including atrial fibrillation, left atrial thrombus, mitral stenosis, mechanical valve, recent myocardial infarction, endocarditis, and so on (29). In case of previous stroke history, patients were classified as recurrent stroke.

A neurologic adjudication of all CIE cases was performed at the time of statistical analysis. For study purposes, three CIE categories were distinguished: CIE before CA, CIE after CA and concomitant CIE and CA diagnoses.

### Statistical Analysis

For all descriptive and inferential analyzes, assumption of normal data distribution was analyzed. Mean and 95% confidence intervals were reported for normally distributed variables and median and min-max range for non-normally distributed variables. Categorical variables were presented as absolute values and percentages. The following tests were used for group comparisons: Student t-test, Wilcoxon-Mann-Whitney test, Chi-square test and Fisher's exact test. The level of statistical significance was set at *p* < 0.05.

Kaplan-Meier and Cox regression analyses were further conducted. Patients having experienced a CIE before CA diagnosis were excluded from the latter. Kaplan-Meier curves described and compared patient survival free from events (death or CIE onset). Mean survival times were computed and difference between curves was assessed with the Log-rank test.

Univariate and multivariate Cox regression models were fitted to assess the independent effect of predictors on time to CIE occurrence. Variables with significant association in univariate analysis (*p* < 0.25) were retained for backward stepwise multivariate regression analysis. Associations were quantified using hazard ratios (HR) and 95% confidence intervals. All statistical analyses were conducted using SAS software 9.4 for Windows (SAS Institute, Cary North Carolina, USA).

## Results

One-hundred and twenty Afro-Caribbean CATTR patients [*N* = 120; 69.2% males, mean age at CA diagnosis: 75.5 ± 7.4 years, median BMI (min-max range): 24.2 (15.9–37.0 kg/m^2^)] were included (Table 1, Supplementary Figure 1). Overall CA population was composed of 17 wild-type ATTR (14.2%), 73 ATTR-V122I (60.8%) and 22 ATTR-I107V (18.3%). CA genotype was undetermined for 8 patients. Patient phenotypes were predominantly cardiac for 52.5% of patients, while 57 patients (47.5%) presented mixed cardiac-neuropathic involvement (Table 1).

**Table 1:** Main characteristics of 120 patients diagnosed with transthyretin cardiac amyloidosis between July 2005 and October 2019.

Characteristics^*^	**All patients (*n* = 120)**
**Clinical variables**	
Age at CA, years	77.1 (55.1–97.2)
Men	83 (69.2)
BMI, kg/m^2^	24.2 (15.9–37.0)
SBP, mmHg	125.0 (80.0–208.0)
DBP, mmHg	77.5 (41.0–111.0)
NYHA class 3–4	31 (25.8)
History of HF episode	60 (50.0)
Chronic renal failure >3	32 (26.7)
History of atrial fibrillation	65 (54.2)
History of heart conduction disorders	40 (33.3)
Peripheral neuropathy	76 (63.9)
**CA characteristics**	
Phenotype - cardiac and neurologic involvement	57 (47.5)
Genotype	
Wild type TTR	17 (14.2)
TTR mutation	95 (79.2)
Type of TTR mutation	
V122I (p.Val142Ile)	73 (60.8)
I107V (p.Ile127Val)	22 (18.3)
**Cerebral Infarction events (CIE)**	36 (30.0)
First-ever CIE	27 (75.0)
Transient ischemic attack	3 (8.3)
Ischemic stroke (permanent neurologic impairment)	33 (91.7)
Cardio-embolic pattern	25 (69.4)
Hemorrhagic transformation	9 (25.0)
Concomitant diagnosis of CA and CIE	16 (44.4)
CIE before CA	6 (16.7)
Interval between CA and CIE, years	4.5 (1.6–12.3)
CIE after CA	14 (38.9)
Interval between CA and CIE, years	2.0 (0.8–4.4)
**Cardiovascular risk factors**	99 (82.5)
Hypertension	80 (66.7)
Diabetes	31 (25.8)
Dyslipidemia	26 (21.7)
Tobacco use	16 (13.3)
Sleep apnea	10 (8.3)
Overweight/obesity	41 (34.2)
**Stroke risk**	
CHA_2_DS_2_-VASc ≥ 3	88 (73.3)
**Electrocardiogram**	
HR, bpm	75.0 (45.0–122.0)
Sinusal rhythm	47 (39.2)
Low voltage QRS	37 (30.8)
**Biological variables**	
Creatinine clearance, mL/min ^n=110^	78.0 (12.0–157.0)
Cardiac troponin hs, ng/L	76.2 (14.8–990.3)
NT-pro BNP, ng/L	3675 (25–84612)
**Echocardiographic variables**	
IVS thickness, mm	17.0 (11.0–31.0)
LVEF, %	47.0 (12.0–82.0)
E/Ea ratio	16.0 (4.0–46.0)
Left atrial enlargement	78 (65.0)
**Treatment**	
Anticoagulant	71 (59.2)
Indication for anticoagulant ^n=65^	
Amyloid cardiopathy	7 (10.8)
Pulmonary embolism	2 (3.1)
Atrial fibrillation	52 (80.0)
Pulmonary artery hypertension	1 (1.5)
Post CIE	2 (3.1)
Deep vein thrombosis	1 (1.5)
Cardiac treatment	99 (82.5)
Diuretics	88 (73.3)
Amiodarone	24 (20.0)
ACE inhibitors	41 (34.2)
Betablockers	29 (24.2)
Digoxine	5 (4.2)

*BMI, Body Mass Index; CA, Cardiac Amyloidosis; CHA2DS2, Congestive heart failure, Hypertension, Age (>65 = 1 point, > 75 = 2 points), Diabetes, previous Stroke/transient ischemic attack (2 points) – VASc, vascular disease (peripheral arterial disease, previous myocardial infarction, aortic atheroma), and sex category (female gender); CIE, Cerebral Ischemic event; NT-pro BNP, N-terminal pro-brain natriuretic peptide; IVS, interventricular septum; LVEF, left ventricular ejection fraction; SBP, Systolic Blood Pressure; DBP, Diastolic Blood Pressure; NYHA, New York Heart Association Functional Classification; HF, Heart Failure; TTR, Transthyretin; ACE, Angiotensin-Converting Enzyme inhibitors; cardiac troponin hs, high sensitivity cardiac troponin*.

* *Results are presented as median (minimum-maximum range) for quantitative variables, and as absolute value (percentage) for categorical variables*.

Thirty-six out of 120 CA patients (30.0%) presented with CIE, including three transient ischemic attacks and 33 permanent ischemic strokes. According to the ASCOD classification, the source of cerebral ischemia was cardioembolism in 25 patients (69.4%). CIE topography was mainly of the anterior circulation type (48.5% of permanent ischemic strokes) (Supplementary Table 1).

**Figure 1:**
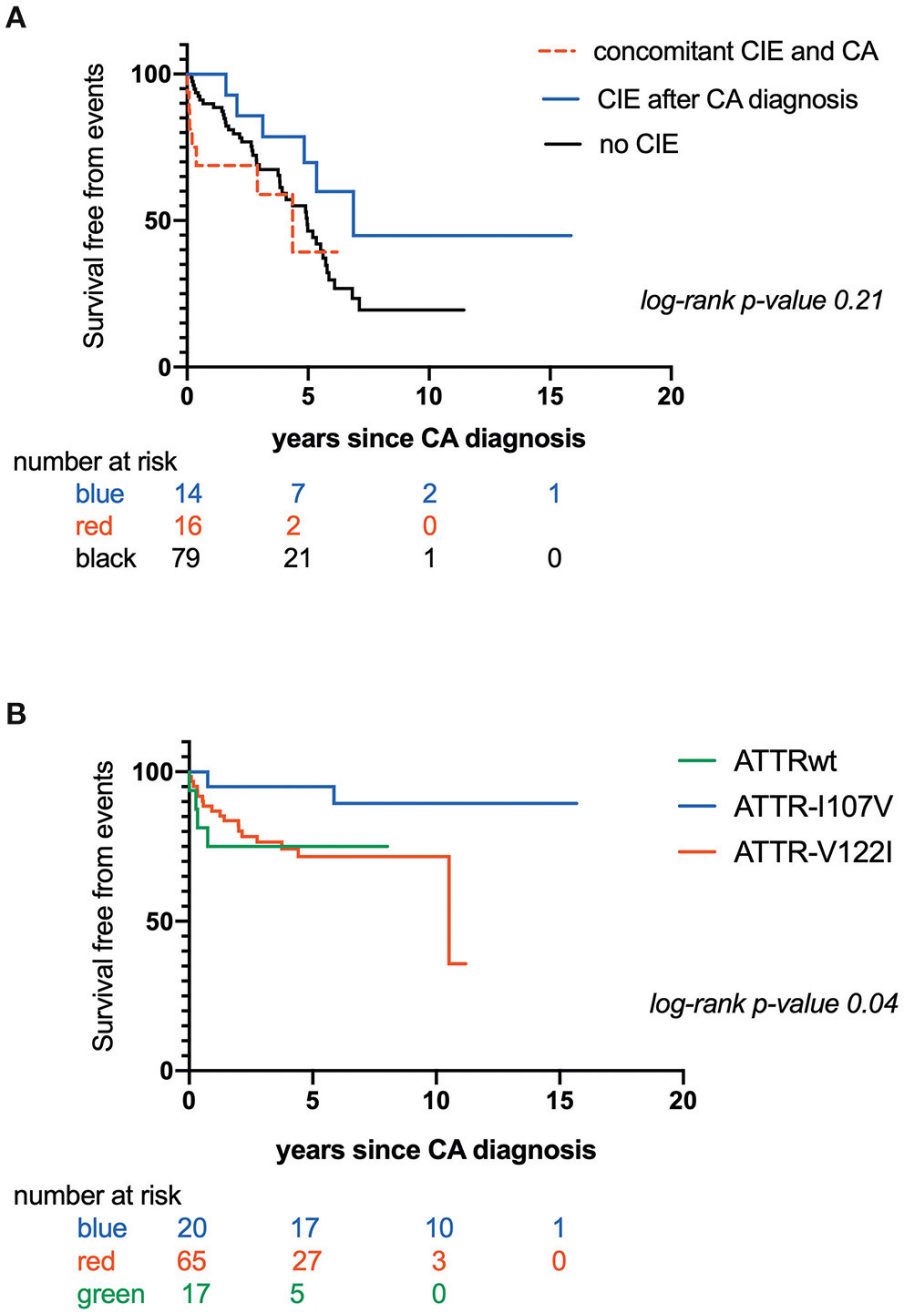
**(A)** Kaplan-Maier analysis of survival free from mortality according to occurrence and timing of Cerebral Ischemic events (CIE); **(B)** Kaplan-Maier analysis of survival free from Cerebral Ischemic events (CIE) according to transthyretin cardiac amyloidosis (CATTR) genotype.

CIE was concomitant with CATTR diagnosis in 16 patients (16/36: 44.4%), while 14 patients (14/36: 38.9 %) experienced CIE over a median CATTR follow-up of 2.0 years (min-max range: 0.8–4.4 years). Six patients (6/36: 16.7%) presented with CIE before CATTR diagnosis. Eleven patients (11/36: 30.6%) experienced recurrent ischemic strokes. Overall mortality was 50.8%. Patients with concomitant diagnosis of CIE and CATTR had the worst outcome, with mean survival of 2.90 ± .5 years after established CA diagnosis, as compared to patients with CIE after CA diagnosis (mean survival: 5.60 ± .6 years) and patients with no CIE (mean survival: 4.40 ± .3 years) (Figure 1A). Table 2 further compares clinical, CA and CIE characteristics according to CA genotype, while patient characteristics with and without CIE are summarized in Table 3.

**Table 2:** Clinical characteristics of the study population according to cardiac amyloidosis genotype (*N* = 112).

Characteristics^*^	**Cardiac amyloidosis genotype**			
	***ATTRwt (n = 17)***	***ATTR-I107V*** ***(n = 22)***	***ATTR-V122I (n= 73)***	***p*** **-value** ^*^
**Clinical variables**				
Age at CA, years	80.4 (69.3–89.6)	68.3 (58.4–78.2)	77.7 (55.1–88.9)	**0.0008**
Men	12 (70.6)	15 (68.2)	51 (69.9)	0.98
BMI, kg/m^2^	25.6 (18.2–32.3)	21.9 (15.9–30.1)	24.3 (17.4–37.0)	0.30
SBP, mmHg	125.0 (89.0–185.0)	130.5 (88.0–180.0)	126.0 (80.0–208.0)	0.55
DBP, mmHg	71.0 (41.0–103.0)	83.5 (57.0–106.0)	78.0 (42.0–111.0)	0.12
NYHA class 3–4	6 (35.3)	1 (4.6)	21 (28.8)	**0.04**
History of decompensated HF	7 (41.2)	4 (18.2)	43 (58.9)	**0.003**
Chronic renal failure >3	4 (23.5)	2 (9.1)	22 (30.1)	0.13
Atrial fibrillation	10 (58.8)	5 (22.7)	45 (61.6)	**0.005**
Heart conduction disorders	6 (35.3)	3 (13.6)	28 (38.4)	0.09
Peripheral neuropathy	6 (35.3)	22 (100.0)	42 (58.3)	**<0.0001**
**CIE characteristics**				
CIE occurrence				0.52
No CIE	12 (70.6)	19 (86.4)	48 (65.8)	
Ischemic stroke with cardioembolic pattern	3 (17.7)	2 (9.1)	17 (23.3)	
Other CIE	2 (11.8)	1 (4.6)	8 (11.0)	
NIHSS ^n=24^				0.51
normal (0)	1 (20.0)	0 (0.0)	2 (11.8)	
minor (1–4)	2 (40.0)	1 (50.0)	7 (41.2)	
moderate (5–14)	2 (40.0)	0 (0.0)	6 (35.3)	
severe (15–20)	0 (0.0)	0 (0.0)	2 (11.8)	
extremely severe (>20)	0 (0.0)	1 (50.0)	0 (0.0)	
CIE timing ^n=33^				0.25
Before CA	0 (0.0)	1 (33.3)	4 (16.0)	
After CA	1 (20.0)	2 (66.7)	11 (44.0)	
Concomitant with CA	4 (80.0)	0 (0.0)	10 (40.0)	
CIE topography ^n=28^				0.58
Anterior circulation	2 (50.0)	2 (66.7)	10 (47.6)	
Posterior circulation	0 (0.0)	0 (0.0)	2 (9.5)	
Lacunar strokes	0 (0.0)	1 (33.3)	1 (4.8)	
Multiple strokes	2 (50.0)	0 (0.0)	8 (38.1)	
**CA characteristics**				
Phenotype - cardiac and neurologic involvement	5 (29.4)	22 (100.0)	26 (36.6)	**<0.0001**
**Cardiovascular risk factors**	14 (87.5)	15 (68.2)	63 (87.5)	0.11
Hypertension	13 (76.5)	10 (45.5)	51 (69.9)	0.07
Diabetes	6 (35.3)	4 (18.2)	18 (24.7)	0.47
Dyslipidemia	3 (17.7)	3 (13.6)	17 (23.3)	0.66
Tobacco use	5 (29.4)	2 (9.1)	9 (12.3)	0.22
Sleep apnea	3 (17.7)	2 (9.1)	5 (6.9)	0.34
Overweight/obesity	9 (52.9)	5 (22.7)	26 (35.6)	0.15
**Stroke risk**				
CHA2DS2-VASc ≥ 3	14 (82.3)	10 (45.5)	57 (78.1)	0.007
**Electrocardiogram**				
HR, bpm	71.0 (45.0–100.0)	73.5 (56.0–97.0)	75.0 (45.0–122.0)	0.92
Sinusal rhythm	6 (35.3)	16 (72.7)	22 (30.1)	**0.002**
Low voltage QRS	6 (66.7)	3 (50.0)	25 (78.1)	0.33
**Biological variables**				
Creatinine clearance, mL/min^n=110^	79.0 (12.0–132.0)	94.0 (22.0–157.0)	74.0 (17.0–129.0)	0.59
Cardiac troponin hs, ng/L	62.0 (14.8–446.5)	54.7 (19.6–990.3)	114.5 (17.1–418.8)	0.09
NT–pro BNP, ng/L	2164 (371–35001)	3179 (115–14413)	4652.5 (25–84612	0.42
**Echocardiographic variables**				
IVS thickness, mm	16.0 (12.0–31.0)	17.0 (12.0–24.0)	17.0 (11.0–30.0)	0.96
LVEF ejection fraction, %	60.0 (40.0–82.0)	51.0 (24.0–71.0)	44.0 (12.0–80.0)	**0.04**
E/Ea ratio	18.0 (11.0–26.0)	13.0 (7.0–22.0)	15.0 (4.0–24.0)	0.59
Left atrial enlargement	14 (82.4)	9 (47.4)	48 (73.9)	**0.04**
**Treatment**				
Anticoagulant	10 (58.8)	7 (31.8)	48 (65.8)	**0.02**
Cardiac treatment	15 (88.2)	16 (72.7)	66 (90.4)	0.12
Diuretics	12 (70.6)	10 (45.5)	59 (80.8)	**0.005**
Amiodarone	3 (17.7)	3 (13.6)	17 (23.3)	0.66
ACE inhibitors	8 (47.1)	9 (40.9)	24 (32.9)	0.49
Betablockers	1 (5.9)	7 (31.8)	18 (24.7)	0.14
Digoxine	0 (0.0)	2 (9.1)	2 (2.7)	0.28

*ATTRwt, wild–type transthyretin amyloidosis; ATTR-I107V, p.Ile127Val transthytein amyloidosis; ATTR-V122I, p.Val142Ile transthytein amyloidosis; CA, Cardiac Amyloidosis; CHA_2_DS_2_, Congestive heart failure, Hypertension, Age (> 65 = 1 point, > 75 = 2 points), Diabetes, previous Stroke/transient ischemic attack (2 points) – VASc, vascular disease (peripheral arterial disease, previous myocardial infarction, aortic atheroma), and sex category (female gender); CIE, Cerebral Ischemic Events; BMI, Body Mass Index; bpm, beat per minute; SBP, Systolic Blood Pressure; DBP, Diastolic Blood Pressure; NIHSS, National Institutes of Health Stroke Scale; NT-pro BNP, N-terminal pro-brain natriuretic peptide; NYHA, New York Heart Association Functional Classification; HF, Heart Failure; IVS, interventricular septum; LVEF left ventricular ejection fraction; TTR, Transthyretin; ACE, Angiotensin-Converting Enzyme inhibitors; cardiac troponin hs, high sensitivity cardiac troponin*.

* *Results are presented as median (minimum-maximum range) for quantitative variables, and as absolute value (percentage) for categorical variables; statistical significance set at p < 0.05. Bold values represent statistically significant p-values (< 0.05)*.

**Table 3:** Clinical characteristics of the study population (*N* = 120) according to presence of Cerebral Ischemic events (CIE).

**Characteristics**	**CIE with cardioembolic pattern**		**No CIE (*n* = 84)**	***p*** **-value** ^**^
	***Yes (n = 25)***	***No*** ^*^ (n = 11)		
**Clinical variables**				
Age at CA, years	75.6 (63.2–88.9)	80.1 (62.3–84.5)	76.7 (55.1–97.2)	0.40
Men	14 (56.0)	8 (72.7)	61 (72.6)	0.28
BMI, kg/m^2^	24.7 (15.9–34.9)	24.4 (18.1–30.5)	23.9 (15.9–37.0)	0.82
SBP, mmHg	125.0 (95.0–165.0)	126.0 (99.0–179.0)	124.5 (80.0–208.0)	0.94
DBP, mmHg	77.0 (54.0–111.0)	79.0 (65.0–101.0)	73.5 (41.0–109.0)	0.58
NYHA class 3–4	7 (28.0)	1 (9.1)	23 (27.4)	0.41
History of HF episode	12 (48.0)	4 (36.4)	44 (52.4)	0.59
Chronic renal failure >3	5 (20.0)	3 (27.3)	24 (28.6)	0.70
Atrial fibrillation	17 (68.0)	7 (63.6)	41 (48.8)	0.19
Heart conduction disorders	10 (40.0)	2 (18.2)	28 (33.3)	0.44
Peripheral neuropathy	14 (56.0)	6 (60.0)	56 (66.7)	0.60
**CA characteristics**				
Phenotype - Cardiac and neurologic involvement	10 (40.0)	4 (40.0)	43 (51.8)	0.50
Genotype				0.92
Wild Type TTR	3 (13.6)	2 (18.2)	12 (15.2)	
TTR mutation	19 (86.4)	9 (81.8)	67 (84.8)	
Type of TTR mutation				0.20
V122I (p.Val142Ile)	17 (89.5)	8 (88.9)	48 (71.6)	
I107V (p.Ile127Val)	2 (10.5)	1 (11.1)	19 (28.4)	
**Cardiovascular risk factors**	20 (80.0)	10 (90.9)	69 (84.2)	0.78
Hypertension	16 (64.0)	9 (81.8)	55 (65.5)	0.53
Diabetes	7 (28.0)	3 (27.3)	21 (25.0)	0.95
Dyslipidemia	7 (28.0)	3 (27.3)	16 (19.1)	0.57
Tobacco use	3 (12.0)	1 (9.1)	12 (14.3)	1.00
Sleep apnea	2 (8.0)	2 (18.2)	6 (7.1)	0.40
Overweight/obesity	8 (32.0)	4 (36.4)	29 (34.5)	0.96
**Stroke risk**				
CHA_2_DS_2_-VASc ≥ 3	24 (96.0)	11 (100.0)	53 (63.1)	**0.0005**
**Electrocardiogram**				
HR, bpm	73.5 (45.0–91.0)	74.5 (52.0–117.0)	77.0 (45.0–122.0)	0.48
Sinusal rhythm,	8 (32.0)	4 (36.4)	35 (41.7)	0.67
Low voltage QRS	7 (28.0)	3 (27.3)	27 (32.1)	0.74
**Biological variables**				
Creatinine clearance, mL/min^n=110^	86.0 (32.0–129.0)	88.0 (46.0–124.0)	72.0 (12.0–157.0)	0.14
Cardiac troponin hs, ng/L	88.4 (19.6–178.0)	63.5 (17.1–153.9)	83.8 (14.8–990.3)	0.78
NT-pro BNP, ng/L	3428.0 (958–13707)	4939.5 (115–28290)	4609.5 (25–84612)	0.23
**Echocardiographic variables**				
IVS thickness, mm	18.5 (12.0–23.0)	16.0 (14.0–25.0)	17.0 (11.0–31.0)	0.49
LVEF, %	52.0 (25.0–80.0)	45.0 (15.0–75.0)	46.0 (12.0–82.0)	0.75
E/Ea ratio	16.0 (7.0–46.0)	10.0 (6.0–24.0)	16.0 (4.0–26.0)	0.36
Left atrial enlargement	21 (87.5)	7 (70.0)	50 (66.7)	0.14
**Treatment**				
Anticoagulant,	21 (84.0)	6 (54.6)	44 (52.4)	**0.02**
Indication for anticoagulant ^n=65^				0.62
Amyloid cardiopathy	3 (15.8)	0 (0.0)	4 (9.8)	
Pulmonary embolism	0 (0.0)	0 (0.0)	2 (4.9)	
Atrial fibrillation	14 (73.7)	5 (100.0)	33 (80.5)	
Pulmonary artery hypertension	0 (0.0)	0 (0.0)	1 (2.4)	
Post CIE	2 (10.5)	0 (0.0)	0 (0.0)	
Deep vein thrombosis	0 (0.0)	0 (0.0)	1 (2.4)	
Cardiac treatment	24 (96.0)	10 (90.9)	70 (83.3)	0.26
Diuretics	20 (80.0)	9 (81.8)	59 (70.2)	0.50
Amiodarone	5 (20.0)	2 (18.2)	17 (20.2)	0.99
ACE inhibitors	8 (32.0)	5 (45.5)	28 (33.3)	0.70
Betablockers	6 (24.0)	2 (18.2)	21 (25.0)	0.88
Digoxine	2 (8.0)	2 (5.6)	3 (3.6)	0.58

*BMI, Body Mass Index; CA, Cardiac Amyloidosis; CHA_2_DS_2_, Congestive heart failure, Hypertension, Age (> 65 = 1 point, > 75 = 2 points), Diabetes, previous Stroke/transient ischemic attack (2 points) – VASc, vascular disease (peripheral arterial disease, previous myocardial infarction, aortic atheroma), and sex category (female gender); CIE, Cerebral Ischemic event; IVS, interventricular septum; LVEF, left ventricular ejection fraction; SBP, Systolic Blood Pressure; DBP, Diastolic Blood Pressure; NT-pro BNP, N-terminal pro-brain natriuretic peptide; NYHA, New York Heart Association Functional Classification; HF, Heart Failure; TTR, Transthyretin; ACE, Angiotensin-Converting Enzyme inhibitors; cardiac troponin hs, high sensitivity cardiac troponin*.

* Results are presented as median (minimum-maximum range) for quantitative variables, and as absolute value (percentage) for categorical variables; *other CIE etiologies (5 small-vessel occlusion; 3 emboli strokes of undetermined source; 3 transient ischemic attacks);

** *statistical significance set at p < 0.05. Bold values represent statistically significant p-values (< 0.05)*.

Of the 36 patients with CIE, five had CATTRwt (5/36: 13.9%), 28 had CATTRv (28/36: 77.8%) and TTR genotype was undetermined in 3 patients (3/36: 8.3%) (Table 3). There were no significant differences in terms of age, gender, body mass index and major cardiovascular characteristics (NYHA, cardiovascular risk factors, history of heart failure, conduction disorders, electrocardiographic and echographic parameters) among patients with or without CIE. Atrial fibrillation (AF) was documented in 24 of the 36 patients with CIE (66.7%) and in 41 patients without CIE (48.8%) (*p* = 0.19). Overall, 71 patients were on an anticoagulation regimen (59.2%). AF was the most frequent indication of anticoagulant treatment in patients with and without CIE (19/27: 70.4% and 33/44: 75.0%, respectively), with higher anticoagulation administration in patients with CIE (*p* = 0.02). It is to be noted that 27 patients (27/71: 38.0%) presented a CIE despite anticoagulation therapy.

**Figure 2:**
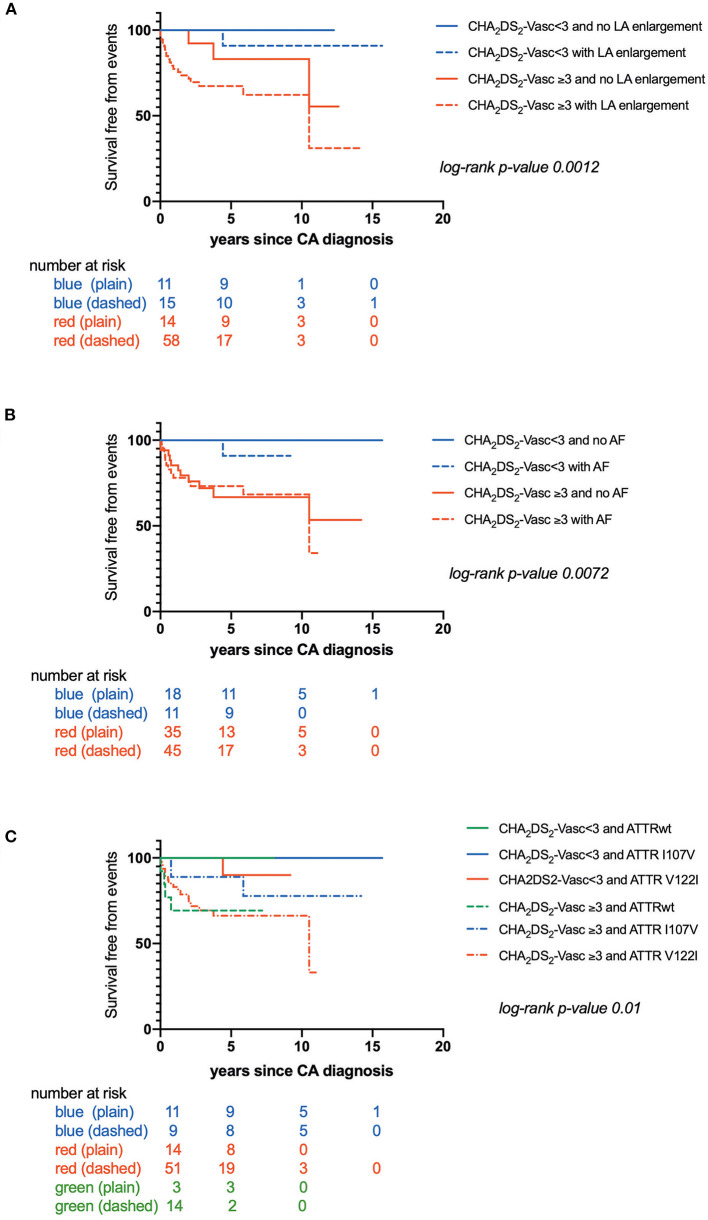
**(A)** Kaplan-Maier analysis of survival free from Cerebral Ischemic events (CIE) according to CHA_2_DS_2_-VASc score and left atrial enlargement; **(B)** Kaplan-Maier analysis of survival free from Cerebral Ischemic events (CIE) according to CHA_2_DS_2_-VASc score and atrial fibrillation; **(C)** Kaplan-Maier analysis of survival free from Cerebral Ischemic events (CIE) according to CHA_2_DS_2_-VASc score and transthyretin cardiac amyloidosis (CATTR) genotype.

CHA_2_DS_2_-VASc score was also higher in patients with CIE (*p* = 0.0005) (Table 3). Kaplan-Meier estimates of CIE risk according to CHA_2_DS_2_-VASc <3 or ≥ 3, LA enlargement and AF are illustrated in Figures 2A,B. Patients with a high CHA_2_DS_2_-VASc (≥ 3) and left atrial enlargement seemed to present with CIE more rapidly after CA diagnosis (mean survival free from event: 6.50 ± .7 years) as compared with a high CHA_2_DS_2_-VASc and no atrial enlargement (mean survival free from event: 9.2 ± 1.0 years) (*p*: 0.0012). The same tendency was observed, in the immediate time frame following CA diagnosis, for patients with a high CHA_2_DS_2_-VASc with or without AF (respective mean survival times free from event of 7.00 ± .7 and 7.30 ± .8 years) (*p*: 0.007)

Furthermore, time to CIE onset was also dependent on CA genotype. Patients with CATTRwt presented with CIE more rapidly (mean survival free from event: 0.60 ± .1 years) as compared to patients expressing the I107V variant (mean survival free from event: 5.60 ± .4 years) or the V122I variant (mean survival free from event: 7.70 ± .6 years) (*p* = 0.04) (Figure 1B). Risk for early CIE onset seemed to follow the same tendency when coupled to a high CHA_2_DS_2_-VASc: mean survival free from event of 0.60 ± .1, 5.30 ± .8, 7.00 ± .7 years respectively for ATTRwt, ATTR-V122I and ATTR-I107V (*p* = 0.01) (Figure 2C).

Univariate Cox regression analysis highlighted a high CHA_2_DS_2_-VASc score (≥3) and left atrial enlargement as potential predictors of CIE in CATTR patients, (Supplementary Tables 2 and 3).

Multivariate analysis retained only a high CHA_2_DS_2_-VASc score (≥3) as an independent predictor of CIE risk (all CIE and CIE with cardioembolic pattern), with Hazard Ratios (95% CI) of respectively 12.03 (1.62–89.24) and 8.24 (1.09-62.56) (Table 4).

**Table 4:** Cox regression analysis of survival free from Cerebral Ischemic Events (CIE) in 114 patients with Transthyretin Cardiac Amyloidosis (multivariate analysis).

**Characteristics**	**Multivariate Cox regression**	
	***HR (95% CI)***	***p-value*** ^*^
Risk of CIE^**^		
Left atrial enlargement	3.29 (0.97–11.12)	0.0554
CHA_2_DS_2_-VASc ≥ 3	12.03 (1.62–89.24)	**0.0150**
		
Risk of CIE with cardioembolic pattern^***^		
Left atrial enlargement	6.41 (0.85–48.53)	0.0719
CHA_2_DS_2_-VASc ≥ 3	8.24 (1.09–62.56)	**0.0414**

*CHA2DS2, Congestive heart failure, Hypertension, Age (> 65 = 1 point, > 75 = 2 points), Diabetes, previous Stroke/transient ischemic attack (2 points) – VASc, vascular disease (peripheral arterial disease, previous myocardial infarction, aortic atheroma), and sex category (female gender); CIE, Cerebral Ischemic event; HR, Hazard ratio; CI, Confidence Interval. Variables included in initial multivariate model, gender, history of atrial fibrillation, CHA2DS2-VASc ≥ 3, CA phenotype, type of TTR mutation, heart rate, left atrial enlargement, cardiac treatment*.

* *Statistical significance set at p < 0.05; goodness-of-fit was assessed (conditions of log-linearity and proportional hazards assumption verified with residual plots) and was found adequate*.

** *N = 114 because of right censoring. We excluded 6 patients for whom CIE occurred before CA diagnosis*.

*** *We excluded 11 patients presenting with other CIE etiologies (5 small-vessel occlusion; 3 emboli strokes of undetermined source; 3 transient ischemic attacks). 5 patients for whom CIE with cardioembolic pattern occurred before CA diagnosis were also excluded (right censoring). Bold values represent statistically significant p-values (< 0.05)*.

## Discussion

Studies addressing the epidemiology of cerebral ischemic events in cardiac amyloidosis (CA) are scarce and mostly limited to case series and observational studies, mainly in Caucasian individuals presenting with AL amyloidosis (16–19, 30–33). Because CATTRv is a rare disease, only few patients are included, even in large multicenter studies evaluating ischemic stroke prevalence in CA patients (8, 9, 13). The present study systematically analyzes information from a large cohort of Afro-Caribbean CATTR patients presenting TTR variants commonly encountered in individuals of identifiable African descent: 60.8% V122I and 18.3% I107V (vs 14.2% ATTRwt) in our sample of 120 CATTR patients. In our series, one third of patients with CATTR experienced cerebral ischemic event (CIE) either concomitantly to CA or during its course. CIE was observed in Afro-Caribbean patients with CATTRv who displayed the typical cardiac and neurological phenotypes associated with V122I and I107V mutations, as generally described in Africans, Black Americans and Afro-Caribbeans (8, 9, 13).

Our study thus highlights that CIE may be frequent and that it may have concomitantly lead to the diagnosis of CATTR in more than 40% of CIE cases (potentially associated with worse patient outcome) in our study sample. Moreover, for 13% of CATTR patients, CIE preceded the diagnosis of systemic amyloidosis by a median interval of 4.5 years. These observations underscore the need to potentially consider early anticoagulation in CATTR patients.

Consistently with previous reports (7, 9, 13), CA patients with V122I TTR variant had more frequently heart failure history and worse myocardial function as compared to CATTRwt and other CATTRv patients. Whereas history of atrial fibrillation and atrial enlargement appeared more frequent in CATTR-V122I and CATTRwt patients, prevalence of CIE was similar independently of CATTR genotype. Survival free from CIE was however shorter in CATTRwt patients, when compared with those with CATTR-I107V and CATTR-V122I.

In our study, CATTR patients who experienced CIE had atrial enlargement and frequently presented at least one episode of permanent AF in their clinical course. Yet, comparison with CATTR patients who did not experience CIE failed to reach statistical significance (*p* = 0.07). Moreover, even if AF was more frequent in patients with CIE, one-third of the latter occurred in CATTR patients with sinus rhythm. These findings are consistent with previous reports attributing these observations to intracardiac thrombi formation along with CA-induced atrial myopathy (14, 18, 19). Proposed mechanisms contributing to intra-cardiac thrombus in CA include blood stasis associated intracavitary turbulence, focal amyloid deposition impairing cardiac wall motion, and amyloid infiltration of coronary arteries leading to myocardial ischemia (14, 18, 19, 34–38). In this context, several studies in CA patients have shown that atrial myopathy can explain thrombi formation in patients with sinus rhythm and/or despite adequate anticoagulation (19, 34, 35). Systemic involvement of the disease, advanced age, multiple cardiovascular risk factors represent other predisposing factors for thromboembolic events.

Overall, our study also readdresses the question of the optimal strategy of anticoagulation therapy in CATTR patients. If anticoagulation must be started as soon as possible in CATTR patients with AF (regardless of CHA_2_DS_2_-VASc score) (35, 36), anticoagulation in CATTR patients in sinus rhythm remains an open question. Chronic anticoagulation is associated with a significantly lower risk for intracardiac thrombosis, with a similar trend observed in CA patients with no AF (35). Effective anticoagulation might thus reduce cerebral ischemic events, which are significant contributors to mortality in CA patients (17). In contrast, anticoagulation may also exacerbate hemorrhagic tendency, which is a well-known complication of amyloidosis attributed to amyloid deposits in vessel walls, potential coexisting coagulopathy or underlying cerebral amyloid angiopathy (CAA) (1).

Furthermore, our results underline that a CHA_2_DS_2_-VASc score ≥ 3 may represent a strong and independent predictive factor of CIE in CATTR patients. In our study sample, this might be linked to age distribution (age range: 55–97 years) as well as a high prevalence of cardiovascular risk factors (hypertension, diabetes) and heart failure. A large series of CA patients has previously identified a likewise association between CIE and a high CHA_2_DS_2_-VASc score, but failed to report its prognostic value in multivariate models due to the small number of CATTR patients in the study sample (18). Overall, a CHA_2_DS_2_-VASc score ≥ 3 may identify CATTR patients with a high risk of CIE, even beyond the presence of AF. Although the benefit of anticoagulation in CATTR patients with no AF remains controversial, a CHA_2_DS_2_-VASc score ≥ 3 could trigger the search for cardiac intracavity thrombi and motivate anticoagulation when applicable.

### Study Strengths and Limitations

This observational study conducted on the French Caribbean island of Martinique is monocentric and retrospective. Even though the present study is one of the largest thus far to address CIE in CATTR patients, the study population was composed only of wild type, V122I and I107V ATTR. Only 36 events were observed, thus possibly resulting in a lack of statistical power to detect other potential factors with moderate effects on thromboembolism, such as cardiovascular risk factors (hypertension, diabetes, dyslipidemia,…). Moreover, the National Institutes of Health Stroke Scale (NIHSS), widely used to assess the severity of acute ischemic stroke, was found to be low in CATTR patients presenting with CIE. This observation may be related to the potential protective role of anticoagulation, which was administered prior to CIE in nearly 60% of CATTR patients. We also attempted to distinguish stroke with a cardioembolic pattern from other ischemic subtypes. However, the study's retrospective nature over a 15-year period might have resulted in uncertain neurologic event identification and adjudication. Furthermore, is spite of the eligibility criteria applied to our study population, we cannot completely rule out cerebral amyloid angiopathy as a potential underlying cause of cerebral ischemia. It is however admitted that strong correlation between CAA and neuroimaging findings allows possible and probable CAA to be diagnosed without pathological specimens (28). On this basis, we excluded all patients presenting with evidence of lobar, cortical and cortical-subcortical intraparenchymal hemorrhage, cerebral microbleeds, and/or cortical superficial siderosis. It is to be noted that in patients with CAA, percutaneous left atrial appendage closure is to be considered as an option for secondary prevention, in place of anticoagulation, due to increased hemorrhagic risk. Lastly, due to our retrospective study design, we were unable to assess the effect of a short time lapse between first onset of CATTR-compatible symptoms and final CATTR diagnosis (indicator of disease aggressiveness) on patient survival free of CIE occurrence. Similarly, we were unable to analyse the effect of novel TTR therapies on CIE risk, as these drugs were authorized only recently on the French Market (after 2019).

## Conclusion

Transthyretin amyloid heart disease could potentially be a full-fledged cause of cerebral ischemic events (mainly cardioembolic). The present study describes one of the largest series of CIE in Afro-Caribbean cardiac amyloidosis patients presenting with V122I and I107V transthyretin variants. We found that CIE occurred in one-third of CATTR patients, and that it seemed to be of worse prognosis when it manifested concomitantly to CATTR diagnosis. A significant proportion of events occurred in patients who had no AF or despite anticoagulation therapy. As reported by Ballantyne et al., thrombotic risk should be better characterized in CATTR patients with integration of echocardiographic parameters and transesophageal echocardiography (14). In addition, our study confirms that a CHA_2_DS_2_-VASc score ≥3 may also be a strong independent predictive factor of CIE in CATTR patients. Further studies are needed to assess the efficacy and timeliness of anticoagulation in CATTR patients, independently of AF.

## Data Availability Statement

The raw data supporting the conclusions of this article will be made available by the authors, without undue reservation.

## Ethics Statement

The study was approved by the institutional review board of the University Hospital of Martinique. All patients were managed in accordance with the amended Declaration of Helsinki (https://www.wma.net/policies-post/wma-declaration-of-helsinki-ethical-principles-for-medical-research-involving-human-subjects/). Written informed consent from the patients/participants or patients/participants' legal guardian/next of kin was not required to participate in this study in accordance with the national legislation and the institutional requirements.

## Author Contributions

RB, T-HT, AM, AS, and JI collected patient data. RB, AS, JI, and RN analyzed and interpreted patient data. RB and RN wrote the manuscript. All authors contributed to the article and approved the submitted version.

## Conflict of Interest

The authors declare that the research was conducted in the absence of any commercial or financial relationships that could be construed as a potential conflict of interest.

## Publisher's Note

All claims expressed in this article are solely those of the authors and do not necessarily represent those of their affiliated organizations, or those of the publisher, the editors and the reviewers. Any product that may be evaluated in this article, or claim that may be made by its manufacturer, is not guaranteed or endorsed by the publisher.

## Abbreviations

AL: Amyloid immunoglobulin light chain
ASCOD A: atherosclerosis
S: small vessel disease
C: cardiac pathology
O: other causes
D: dissection
CA: Cardiac amyloidosis
CATTR: cardiac transthyretin amyloidosis
CHA_2_DS_2_-VASc: Congestive heart failure; Hypertension; Age; Diabetes; Stroke - Vascular Age; Sex
CT: Computed tomography
CIE: Cerebral ischemic event
ENMG: Electroneuromyogram
FLAIR: Fluid attenuation inversion recovery
HMDP: Hydroxy methylene diphosphonate
Ile: Isoleucine
IRB: Institutional review board
MRI: Magnetic resonance imaging
ATTRv: mutated transthyretin amyloidosis
TIA: Transient ischemic attacks
TTR: transthyretin
TTRv: transthyretin variant
Val: Valine
ATTRwt: wild type transthyretin amyloidosis.

## References

[1] Wechalekar AD Gillmore JD Hawkins PN. Systemic amyloidosis. Lancet. (2016) 387:2641–54. 10.1016/S0140-6736(15)01274-X 26719234

[2] Gertz MA Dispenzieri A Sher T. Pathophysiology and treatment of cardiac amyloidosis. Nat Rev Cardiol. (2015) 12:91–102. 10.1038/nrcardio.2014.165 25311231

[3] Maurer MS Hanna M Grogan M Dispenzieri A Witteles R Drachman B. Genotype and phenotype of transthyretin cardiac amyloidosis: THAOS (Transthyretin Amyloid Outcome Survey). J Am Coll Cardiol. (2016) 68:161–72. 10.1016/j.jacc.2016.03.596 27386769 PMC4940135

[4] Ruberg FL Grogan M Hanna M Kelly JW Maurer MS. Transthyretin amyloid cardiomyopathy: JACC state-of-the-art review. J Am Coll Cardiol. (2019) 73:2872–91. 10.1016/j.jacc.2019.04.003 31171094 PMC6724183

[5] Buxbaum JN Ruberg FL. Transthyretin V122I (pV142I)^*^ cardiac amyloidosis: an age-dependent autosomal dominant cardiomyopathy too common to be overlooked as a cause of significant heart disease in elderly African Americans. Genet Med. (2017) 19:733–42. 10.1038/gim.2016.200 28102864 PMC5509498

[6] Dungu J Sattianayagam PT Whelan CJ Gibbs SD Pinney JH Banypersad SM. The electrocardiographic features associated with cardiac amyloidosis of variant transthyretin isoleucine 122 type in Afro-Caribbean patients. Am Heart J. (2012) 164:72–9. 10.1016/j.ahj.2012.04.013 22795285

[7] Dungu JN. Cardiac amyloid - an update. Eur Cardiol. (2015) 10:113–7. 10.15420/ecr.2015.10.2.113 30310435 PMC6159448

[8] Dungu JN Papadopoulou SA Wykes K Mahmood I Marshall J Valencia O. Afro-Caribbean heart failure in the United Kingdom: cause, outcomes, and ATTR V122I cardiac amyloidosis. Circ Heart Fail. (2016) 9:e003352. 10.1161/CIRCHEARTFAILURE.116.003352 27618855

[9] Goyal A Lahan S Dalia T Ranka S Bhattad VB Patel RR. Clinical comparison of V122I genotypic variant of transthyretin amyloid cardiomyopathy with wild-type and other hereditary variants: a systematic review. Heart Fail. Rev. (2021). 10.1007/s10741-021-10098-6 33768376

[10] Inamo J Ozier-Lafontaine N Atallah A Merle S Banydeen R Neviere R. Frequency of cardiac amyloidosis in patients with unexplained left ventricular hypertrophy: the Caribbean Amyloidosis Study. Arch Cardiovasc Dis. (2018) 10:29. 10.1016/j.acvdsp.2017.11.055

[11] Monfort A Banydeen R Demoniere F Courty B Codiat R Neviere R. Restrictive cardiac phenotype as primary cause of impaired aerobic capacity in Afro-Caribbean patients with val122ile variant transthyretin amyloid cardiomyopathy. Amyloid. (2020) 27:145–52. 10.1080/13506129.2020.1722098 32024385

[12] Oliveira Da Silva L Fabre J Monfort A Villeret J Citony I Cohen-Tenoudji P. ‘Green Apple’ Heart Failure. West Indian Med J. (2014) 63:673–5. 10.7727/wimj.2013.255 25803389 PMC4663962

[13] Shah KB Mankad AK Castano A Akinboboye OO Duncan PB Fergus IV. Transthyretin Cardiac Amyloidosis in Black Americans. Circ Heart Fail. (2016) 9:e002558. 10.1161/CIRCHEARTFAILURE.115.002558 27188913 PMC4874558

[14] Smith EE Charidimou A Ayata C Werring DJ Greenberg SM. Cerebral amyloid angiopathy-related transient focal neurologic episodes. Neurology. (2021) 97:231–8. 10.1212/WNL.0000000000012234 34016709 PMC8356377

[15] Sousa L Coelho T Taipa R CNS involvement in hereditary transthyretin amyloidosis. Neurology. (2021) 97:1111–9. 10.1212/WNL.0000000000012965 34663645

[16] Zhang XD Liu YX Yan XW Fang LG Fang Q Zhao DC. Cerebral embolism secondary to cardiac amyloidosis: A case report and literature review. Exp Ther Med. (2017) 14:6077–83. 10.3892/etm.2017.5301 29250142 PMC5729392

[17] Zubkov AY Rabinstein AA Dispenzieri A Wijdicks EF. Primary systemic amyloidosis with ischemic stroke as a presenting complication. Neurology. (2007) 69:1136–41. 10.1212/01.wnl.0000276951.39112.2b 17846413

[18] Cappelli F Tini G Russo D Emdin M Del Franco A Vergaro G. Arterial thrombo-embolic events in cardiac amyloidosis: a look beyond atrial fibrillation *Amyloid*. (2021) 28:12–8. 10.1080/13506129.2020.1798922 32981389

[19] Feng D Edwards WD Oh JK Chandrasekaran K Grogan M Martinez MW. Intracardiac thrombosis and embolism in patients with cardiac amyloidosis. Circulation. (2007) 116:2420–6. 10.1161/CIRCULATIONAHA.107.697763 17984380

[20] Yamano M Azuma A Yazaki M Ikeda S Sawada T Matsubara H. Early cardiac involvement in senile systemic amyloidosis: a case report. Amyloid. (2008) 15:54–9. 10.1080/13506120701815506 18266122

[21] Selvaraj S Claggett B Minamisawa M Windham BG Chen LY Inciardi RM. Atrial Fibrillation and Ischemic Stroke With the Amyloidogenic V122I Transthyretin Variant Among Black Americans. J Am Coll Cardiol. (2021) 78:89–91. 10.1016/j.jacc.2021.04.042 33957237 PMC11391793

[22] Jacobson DR Alexander AA Tagoe C Garvey WT Williams SM Tishkoff S. The prevalence and distribution of the amyloidogenic transthyretin (TTR) V122I allele in Africa. Mol Genet Genomic Med. (2016) 4:548–56. 10.1002/mgg3.231 27652282 PMC5023940

[23] Jacobson DR Alexander AA Tagoe C Buxbaum JN. Prevalence of the amyloidogenic transthyretin (TTR) V122I allele in 14 333 African-Americans. Amyloid. (2015) 22:171–4. 10.3109/13506129.2015.1051219 26123279

[24] Monplaisir N Valette I Lepage V Dijon V Lavocat E Ribal C. Study of HLA antigens of the Martinican population. Tissue Antigens. (1985) 26:1–11. 10.1111/j.1399-0039.1985.tb00928.x 3862264

[25] Perugini E Guidalotti PL Salvi F Cooke RM Pettinato C Riva L. Non-invasive etiologic diagnosis of cardiac amyloidosis using 99mTc-3,3-diphosphono-1,2-propanodicarboxylic acid scintigraphy. J Am Coll Cardiol. (2005) 46:1076–84. 10.1016/j.jacc.2005.05.073 16168294

[26] Kolominsky-Rabas PL Weber M Gefeller O Neundoerfer B Heuschmann PU. Epidemiology of ischemic stroke subtypes according to TOAST criteria: incidence, recurrence, and long-term survival in ischemic stroke subtypes: a population-based study. Stroke. (2001) 32:2735–40. 10.1161/hs1201.100209 11739965

[27] El-Koussy M Schroth G Brekenfeld C Arnold M. Imaging of acute ischemic stroke. Eur Neurol. (2014) 72:309–16. 10.1159/000362719 25323674

[28] Chen D Roytman M Kirou KA Navi BB Schweitzer AD, A case of inflammatory cerebral amyloid angiopathy after ischemic stroke - a potential risk factor related to blood-brain barrier disruption. Clin Imaging. (2022) 82:161–5. 10.1016/j.clinimag.2021.11.007 34847499

[29] Yang XL Zhu DS Lv HH Huang XX Han Y Wu S. Etiological Classification of Cerebral Ischemic Stroke by the TOAST, SSS-TOAST, and ASCOD Systems: The Impact of Observer's Experience on Reliability. Neurologist. (2019) 24:111–4. 10.1097/NRL.0000000000000236 31246719

[30] Botker HE Rasmussen OB. Recurrent cerebral embolism in cardiac amyloidosis. Int J Cardiol. (1986) 13:81–3. 10.1016/0167-5273(86)90082-3 3771004

[31] Cho KH Cho YM Kim JS. Embolic infarction associated with cardiac amyloidosis. J Clin Neurol. (2005) 1:92–6. 10.3988/jcn.2005.1.1.92 20396476 PMC2854936

[32] Marques P Beato-Coelho J Durães J Geraldo A. Ischaemic stroke as the initial manifestation of systemic amyloidosis. BMJ case rep. (2019) 12:e228979. 10.1136/bcr-2018-228979 31256048 PMC6605910

[33] Suleiman S Coughlan JJ Moore D. Cardiac amyloidosis presenting with recurrent ischaemic strokes. BMJ case rep. (2020) 13:e231910. 10.1136/bcr-2019-231910 32094234 PMC7046391

[34] Ballantyne B Manian U Sheyin O Davey R De S. Stroke risk and atrial mechanical dysfunction in cardiac amyloidosis. ESC Heart Fail. (2020) 7:705–7. 10.1002/ehf2.12602 31965737 PMC7160485

[35] Feng D Syed IS Martinez M Oh JK Jaffe AS Grogan M. Intracardiac thrombosis and anticoagulation therapy in cardiac amyloidosis. Circulation. (2009) 119:2490–7. 10.1161/CIRCULATIONAHA.108.785014 19414641

[36] Mitrani LR De Los Santos J Driggin E Kogan R Helmke S Goldsmith J. Anticoagulation with warfarin compared to novel oral anticoagulants for atrial fibrillation in adults with transthyretin cardiac amyloidosis: comparison of thromboembolic events and major bleeding. Amyloid. (2021) 28:30–4. 10.1080/13506129.2020.1810010 32814468 PMC8018530

[37] Henein MY Suhr OB Arvidsson S Pilebro B Westermark P Hörnsten R. Reduced left atrial myocardial deformation irrespective of cavity size: a potential cause for atrial arrhythmia in hereditary transthyretin amyloidosis. Amyloid. (2018) 25:46–53. 10.1080/13506129.2018.1430027 29369708

[38] Martinez-Naharro A Gonzalez-Lopez E Corovic A Mirelis JG Baksi AJ Moon JC. High Prevalence of Intracardiac Thrombi in Cardiac Amyloidosis. J Am Coll Cardiol. (2019) 73:1733–4. 10.1016/j.jacc.2019.01.035 30947929

