# Supplementary material for: Cerebral Ischemic Events: An Overlooked Complication of Transthyretin Cardiac Amyloidosis in Afro-Caribbean Patients
Source: Front Neurol. 2022 May 19;13:878292. doi: 10.3389/fneur.2022.878292 (PMC9161261; doi:10.3389/fneur.2022.878292)
Supplement: Supplementary file 1 [file Table_1.docx]

**Supplemental Table 1 – Main characteristics of Cerebral Ischemic Events (CIE) in 120 patients diagnosed with transthyretin cardiac amyloidosis between** **July 2005 and October 2019. University Hospital of Martinique**

| **Characteristics** | **Parameters** |
| --- | --- |
| **Cerebral Ischemic events (CIE), n (%)** | **36 (30.0**) |
| First-ever CIE, n (%) | 27 (75.0) |
| Transient ischemic attack, n (%) | 3 (8.3) |
| Ischemic stroke (permanent neurologic impairment), n (%) | 33 (91.7) |
| Cardio-embolic pattern | 25 (75.8) |
| Small-vessel occlusions (lacunar strokes) | 5 (15.2) |
| Cryptogenic (ESUS) | 3 (9.1) |
| Age at first CIE diagnosis, years |  |
| Mean ± std | 74.5 ± 8.3 |
| Median (min-max) | 74.8 (48.6 - 88.8) |
| Number of CIE / patient |  |
| Mean ± std | 1.5 ± 1.1 |
| Median (min-max) | 1.0 (1.0-6.0) |
| Topography, n (%) |  |
| Anterior circulation |  |
| Partial Anterior Circulation Stroke (PACS) | 13 (36.1) |
| Total Anterior Circulation Stroke (TACS) | 1 (2.8) |
| Junctional Stroke | 2 (5.6) |
| Posterior circulation |  |
| Left posterior cerebral artery | 2 (5.6) |
| Lacunar Stroke (LACS) | 2 (5.6) |
| Multiple Strokes | 11 (30.6) |
| NIHSS score, n (%) |  |
| normal (0) | 4 (11.1) |
| minor (1-4) | 10 (27.8) |
| moderate (5-14) | 8 (22.2) |
| severe (15-20) | 4 (11.1) |
| extremely severe (>20) | 1 (2.8) |
| Concomitant diagnosis of CA and CIE, n (%) | 16 (44.4) |
| CIE before CA, n (%) | 6 (16.7) |
| Interval between CA and CIE, years |  |
| mean ± std | 5.4 ± 4.0 |
| median interval (min-max) | 4.5 [1.6 - 12.3] |
| CIE after CA, n (%) | 14 (38.9) |
| Interval between CA and CIE, years |  |
| mean ± std | 2.1 ± 1.1 |
| median interval (min-max) | 2.0 [0.8 - 4.4] |

Abbreviations: CA: Cardiac Amyloidosis; CIE: Cerebral Ischemic Events; std: standard deviation; NIHSS: National Institutes of Health Stroke Scale; ESUS: Embolic Stroke of Undetermined Source

**Supplemental Table 2: Cox regression analysis of survival free from Cerebral Ischemic events (CIE) in 114 patients with Transthyretin Cardiac Amyloidosis** (univariate analysis)*

| **Characteristics** | **Univariate Cox regression** | | |
| --- | --- | --- | --- |
|  | *HR (95% CI)* | *p-value *** |  |
| **Clinical variables** |  |  |  |
| Age, years | 1.01 (0.97-1.06) | 0.59 |  |
| Female gender | 1.60 (0.74-3.42) | **0.23** |  |
| BMI | 1.02 (0.93-1.12) | 0.72 |  |
| SBP, mmHg | 1.00 (0.99-1.02) | 0.76 |  |
| DBP, mmHg | 1.02 (0.99-1.04) | 0.31 |  |
| NYHA class 3-4 | 0.99 (0.42-2.34) | 0.98 |  |
| History of HF episode | 0.70 (0.34-1.48) | 0.35 |  |
| Chronic renal failure >3 | 0.82 (0.35-1.91) | 0.64 |  |
| History of atrial fibrillation | 1.63 (0.78-3.44) | **0.20** |  |
| CHA_2_DS_2_-VASc ≥ 3 | 13.69 (1.86-100.64) | **0.01** |  |
|  |  |  |  |
| **Cardiac amyloidosis** |  |  |  |
| Phenotype |  | **0.07** |  |
| Cardiac + neurologic | 0.49 (0.23-1.06) |  |  |
| Cardiac only | reference |  |  |
| Presence of TTR mutation | 0.71 (0.27-1.90) | 0.50 |  |
| Type of TTR mutation |  | **0.07** |  |
| ATTR-V122I | 0.92 (0.34-2.45) |  |  |
| ATTR-I107V | 0.16 (0.03-0.90) |  |  |
| ATTRwt | reference |  |  |
|  |  |  |  |
| **Electrocardiogram** |  |  |  |
| HR, bpm | 0.98 (0.96-1.01) | **0.19** |  |
| Sinus rhythm | 0.86 (0.41-1.81) | 0.69 |  |
| Microvoltage | 1.22 (0.31-4.80) | 0.78 |  |
|  |  |  |  |
| **Biological variables** |  |  |  |
| Creatinine clearance (mL/min) | 1.00 (0.99-1.01) | 0.71 |  |
|  |  |  |  |
| **Echocardiographic variables** |  |  |  |
| Ejection fraction, % | 2.46 (0.24-25.27) | 0.45 |  |
| Left atrial enlargement | 3.80 (1.14-12.63) | **0.03** |  |
|  |  |  |  |
| **Treatments** |  |  |  |
| Cardiac treatment | 2.51 (0.60-10.55) | **0.21** |  |

Abbreviations: ATTR-wt: wild-type transthyretin amyloidosis; ATTR-I107V: p.Ile127Val transthyretin amyloidosis; ATTR-V122I: p.Val142Ile transthyretin amyloidosis; bpm: beat per minute; CHA2DS2: Congestive heart failure, Hypertension, Age ( > 65 = 1 point, > 75 = 2 points), Diabetes, previous Stroke/transient ischemic attack (2 points) – VASc: vascular disease (peripheral arterial disease, previous myocardial infarction, aortic atheroma), and sex category (female gender); CIE: Cerebral Ischemic Events; BMI: Body Mass Index; SBP: Systolic Blood Pressure; DBP: Diastolic Blood Pressure; NYHA: New York Heart Association Functional classification; HF: Heart Failure; TTR: Transthyretin; HR: Heart rate; HR: Hazard ratio; CI: Confidence Interval.

* N=114 because of right censoring. We excluded 6 patients for whom CIE occurred before CA diagnosis

** statistical significance set at p<0.25

**Supplemental Table 3: Cox regression analysis of survival free from Cerebral Ischemic Events (CIE) with Cardioembolic pattern in 104 patients with Transthyretin Cardiac Amyloidosis** (univariate analysis)*

| **Characteristics** | **Univariate Cox regression** | | |
| --- | --- | --- | --- |
|  | *HR (95% CI)* | *p-value *** |  |
| **Clinical variables** |  |  |  |
| Age, years | 1.01 (0.95-1.08) | 0.67 |  |
| Female gender | 2.03 (0.82-4.98) | **0.12** |  |
| BMI | 1.01 (0.90-1.14) | 0.83 |  |
| SBP, mmHg | 1.00 (0.98-1.02) | 0.93 |  |
| DBP, mmHg | 1.01 (0.98-1.04) | 0.60 |  |
| NYHA class 3-4 | 1.65 (0.65-4.19) | 0.30 |  |
| History of HF episode | 0.84 (0.35-2.06) | 0.71 |  |
| Chronic renal failure >3 | 0.86 (0.31-2.36) | 0.77 |  |
| Atrial fibrillation | 1.61 (0.64-4.02) | 0.31 |  |
| Heart conduction disorders | 1.28 (0.52-3.14) | 0.59 |  |
| CHA_2_DS_2_-VASc ≥ 3 | 9.80 (1.31-73.32) | **0.03** |  |
|  |  |  |  |
| **Cardiac amyloidosis** |  |  |  |
| Phenotype |  | **0.10** |  |
| Cardiac and neurologic | 0.45 (0.18-1.15) |  |  |
| Cardiac only | reference |  |  |
| Presence of TTR mutation | 0.79 (0.23-2.77) | 0.72 |  |
| Type of TTR mutation |  | **0.14** |  |
| ATTR-V122I | 1.06 (0.30-3.70) |  |  |
| ATTR-I107V | 0.13 (0.01-1.36) |  |  |
| ATTRwt | reference |  |  |
|  |  |  |  |
| **Electrocardiogram** |  |  |  |
| HR, bpm | 0.97 (0.94-1.01) | **0.12** |  |
| Sinus Rhythm | 0.89 (0.36-2.22) | 0.81 |  |
| Microvoltage | 0.93 (0.22-3.98) | 0.92 |  |
|  |  |  |  |
| **Biological variables** |  |  |  |
| Creatinine clearance (mL/min) | 1.00 (0.99-1.01) | 0.97 |  |
|  |  |  |  |
| **Echocardiographic variables** |  |  |  |
| LV ejection fraction, % | 3.58 (0.21-60.15) | 0.38 |  |
| Left atrial enlargement | 7.88 (1.05-59.14) | **0.04** |  |
|  |  |  |  |
| **Treatments** |  |  |  |
| Cardiac treatment | 3.40 (0.45-25.50) | **0.23** |  |

Abbreviations: ATTR-wt: wild-type transthyretin amyloidosis; ATTR-I107V: p.Ile127Val transthyretin amyloidosis; ATTR-V122I: p.Val142Ile transthyretin amyloidosis; CHA_2_DS_2_: Congestive heart failure, Hypertension, Age ( > 65 = 1 point, > 75 = 2 points), Diabetes, previous Stroke/transient ischemic attack (2 points) – VASc: vascular disease (peripheral arterial disease, previous myocardial infarction, aortic atheroma), and sex category (female gender); CIE: Cerebral Ischemic Events; BMI: Body Mass Index; SBP: Systolic Blood Pressure; DBP: Diastolic Blood Pressure; HF: Heart Failure; NYHA: New York Heart Association Functional classification; LV left ventricular; TTR: Transthyretin; HR: Heart rate; HR: Hazard ratio; CI: Confidence Interval.

* We excluded 11 patients presenting with other CIE etiologies (5 small-vessel occlusion; 3 emboli strokes of undetermined source; 3 transient ischemic attacks). 5 patients for whom CIE with cardioembolic pattern occurred before CA diagnosis were also excluded (right censoring); ** statistical significance set at p<0.25
