# Supplementary figures and images for: Cerebral Ischemic Events: An Overlooked Complication of Transthyretin Cardiac Amyloidosis in Afro-Caribbean Patients
Source: Front Neurol. 2022 May 19;13:878292. doi: 10.3389/fneur.2022.878292 (PMC9161261; doi:10.3389/fneur.2022.878292)

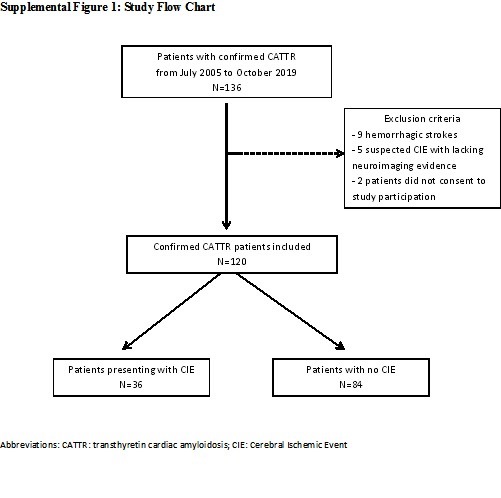

Supplement: Supplementary file 2 [file Image_1.jpeg]
